# Activity of andrographolide against chikungunya virus infection

**DOI:** 10.1038/srep14179

**Published:** 2015-09-18

**Authors:** Phitchayapak Wintachai, Parveen Kaur, Regina Ching Hua Lee, Suwipa Ramphan, Atichat Kuadkitkan, Nitwara Wikan, Sukathida Ubol, Sittiruk Roytrakul, Justin Jang Hann Chu, Duncan R. Smith

**Affiliations:** 1 Institute of Molecular Biosciences, Mahidol University, Bangkok, Thailand; 2 Department of Microbiology, Yong Loo Lin School of Medicine, National University of Singapore, Singapore; 3 Department of Microbiology, Faculty of Science, Mahidol University, Bangkok, Thailand; 4 Center for Emerging and Neglected Infectious Diseases, Mahidol University, Bangkok, Thailand; 5 National Center for Genetic Engineering and Biotechnology (BIOTEC), National Science and Technology Development Agency, Pathum Thani, Thailand

## Abstract

Chikungunya virus (CHIKV) is a re-emerging mosquito-borne alphavirus that has recently engendered large epidemics around the world. There is no specific antiviral for treatment of patients infected with CHIKV, and development of compounds with significant anti-CHIKV activity that can be further developed to a practical therapy is urgently required. Andrographolide is derived from *Andrographis paniculata*, a herb traditionally used to treat a number of conditions including infections. This study sought to determine the potential of andrographolide as an inhibitor of CHIKV infection. Andrographolide showed good inhibition of CHIKV infection and reduced virus production by approximately 3log_10_ with a 50% effective concentration (EC_50_) of 77 μM without cytotoxicity. Time-of-addition and RNA transfection studies showed that andrographolide affected CHIKV replication and the activity of andrographolide was shown to be cell type independent. This study suggests that andrographolide has the potential to be developed further as an anti-CHIKV therapeutic agent.

Chikungunya virus (CHIKV), an arthropod-borne virus, is the member of genus *Alphavirus*, family *Togaviridae* and is the causative agent of chikungunya fever (CHIKF). CHIKV has recently re-emerged and caused large epidemics in Africa, India and Southeast Asia between 2004 and 2010^1^,^2^,^3^,^4^ and more recently in the Americas starting from 2014^5^,^6^. CHIKV is an approximately 70 nm enveloped virus with an icosahedral nucleocapsid. The genome is an 11.8 kb positive single stranded RNA, containing a 5′-methylguanylate cap and a 3′-poly A tail with two open reading frames encoding for four nonstructural proteins (nsP1-nsP4) in the first, 5′ open reading frame and three structural proteins (capsid, E1 and E2) and two protein of unidentified function (E3 and 6 K) in the second, 3′ reading frame^7^.

CHIKV is transmitted to humans by the bite of infected *Aedes* (*Ae*.) mosquitoes and it was first isolated in Tanzania, Africa during an outbreak in 1952^8^,^9^. Subsequently, CHIKV was identified as the causative agent in other febrile disease outbreaks in Africa and elsewhere, and phylogenic analysis identified three CHIKV lineages, the East, Central and South African (ECSA) lineage, the West African lineage and the Asian lineage^10^. In 2004, an outbreak of CHIKV occurred in Kenya, and the causative virus of the ECSA lineage subsequently spread to the islands of the Indian Ocean and then to India and Southeast Asia^3^,^11^. In outbreaks in the Indian Ocean island of Reunion, and subsequently in India, it was observed that while the initial wave of infections was driven by transmission in *Ae*. *aegypti* mosquitoes, later, more severe waves of transmission were driven by transmission in *Ae*. *albopictus*^12^,^13^. It was subsequently shown^14^,^15^ that transmission in *Ae*. *albopictus* mosquitoes was facilitated by the emergence of strains of CHIKV with the substitution of a valine instead of the normal alanine at position 226 of the CHIKV E1 protein (E1 A226V). Remarkably, the E1 A226V substitution was shown to have occurred independently several times^14^,^16^.

CHIKV infected patients develop symptoms within 3–7 days of being bitten by an infected mosquito^7^. The clinical symptoms of infection where manifested show great similarity with dengue fever, and include a sudden febrile illness, rash, headache, edema of the extremities, gastrointestinal complaints and myalgia^17^. Additionally polyarthralgia, which frequently persists for two or more months, is considered a hallmark of CHIKV infection^18^. There is currently no specific treatment for CHIKV infection, and treatment is primarily symptomatic. Chikungunya fever is seldom fatal, although increased neurological involvement^19^,^20^ and occasional fatalities have been reported^21^,^22^. However the disease, and particularly the persistent arthralgia can have long term impact on an infected person’s quality of life, particularly through disruption of their ability to work^23^. Specific antivirals that could reduce or eliminate CHIKV, either as a treatment or as a prophylactic in epidemic situations, are urgently required.

*Andrographis paniculata* (Burm. f.) Nees (also known as the “king of bitters”) is a traditional medicinal plant used to treat infections and other disorders^24^. It is originally believed to be native to India and Sri Lanka, but is now commonly found and cultivated around much of Southern and Southeastern Asia. Andrographolide, a bicyclic diterpenoid lactone is believed to be the main bioactive ingredient with potential anti-inflammatory and hepatoprotective effects^25^. Given the traditional usage of *Andrographis paniculata* to treat infections, this study sought to determine whether the principal bioactive ingredient (andrographolide) possessed detectable anti-CHIKV activities. The cell line selected for investigation was the liver cell line HepG2 which has been shown to be highly susceptible to CHIKV^26^ and liver involvement in the disease has been shown in mouse^27^ and non human primate model systems^28^. In addition a non-relevant cell line previously used in evaluating anti-CHIKV compounds^29^, BHK-21, was investigated. Remarkably, andrographolide showed good potency in inhibiting CHIKV replication with no marked cytotoxicity in a cell culture system. These results suggest that andrographolide has significant potential for further development as an anti-CHIKV agent.

## Results

### Evaluation of cytotoxicity of andrographolide

Cytotoxicity of andrographolide in the range 1–100 μM was evaluated twice independently using the MTT assay at 24 hrs post-treatment (Supplemental Figure S1) and the alamarBlue assay at 24, 36 and 48 hr post treatment (Supplemental Figure S2). No significant cytotoxicity was seen at 24 and 36 hrs post treatment, but some effect was seen on cell viability at 48 hrs post treatment (Supplemental Figure 2). The 50% cytotoxic concentrations calculated from the dose response curves were 1098 μM, 1024 μM and 179 μM at 24, 36 and 48 hrs.

### Evaluation of virucidal activity of andrographolide

Stock CHIKV (E1:226VT) was incubated with andrographolide (1, 50 and 100 μM) or with medium only or vehicle only as controls for 1 hr at 37 °C after which the virus titer was determined directly by standard plaque assay. No reduction in virus titer was seen (Supplemental Figure S3), suggesting that andrographolide does not have a direct virucidal activity. This result was confirmed with longer incubation of andrographolide with CHIKV, and no effect was seen even with 12 hr incubation prior to infection (Supplemental Figure S4).

### Effect of andrographolide on CHIKV infection

**Figure 1:**
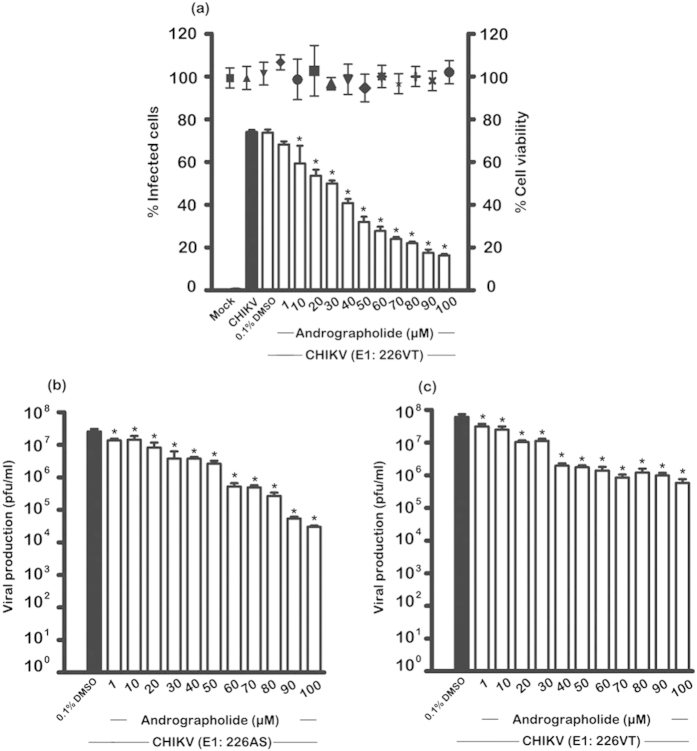
Effect of andrographolide on CHIKV infection. HepG2 cells were pre-treated with varying concentrations of andrographolide, or with vehicle only, or not treated and then infected or mock infected with (**a**) CHIKV E1: 226VT (**b**) CHIKV E1: 226AS and (**c**) E1: 226VT followed by incubation under standard conditions in the presence or absence of the drug or vehicle as appropriate. At 24 h.p.i. the level of (**a**) infection and cell viability were determined by flow cytometry and trypan blue staining or (**b**,**c**) viral production determined by standard plaque assay. Experiments were undertaken independently in triplicate, with duplicate plaque assay. Bars show mean +/− SD (*; *p* value < 0.05).

**Figure 2:**
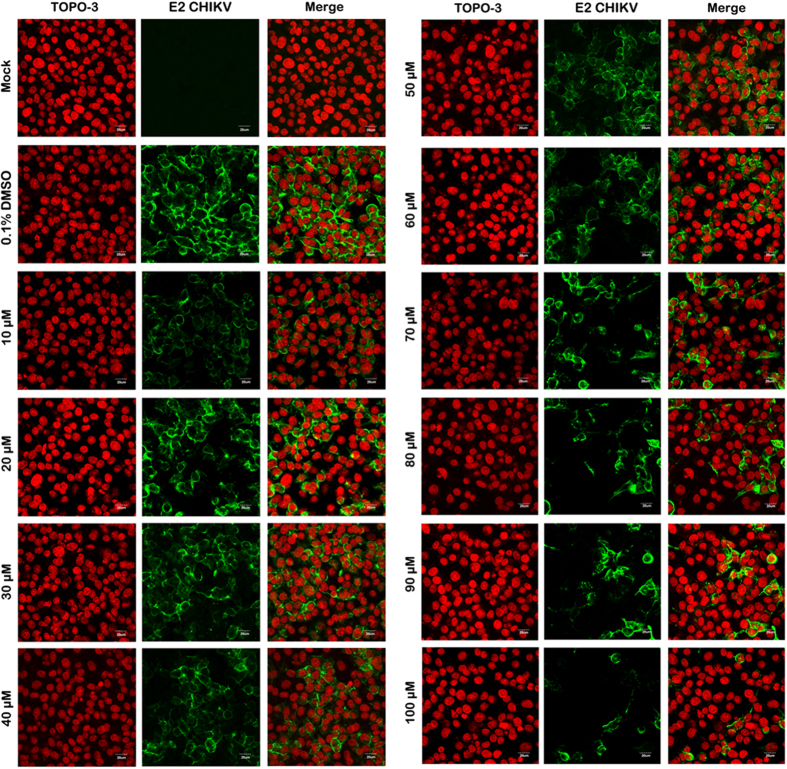
Effect of andrographolide treatment on CHIKV infection as assessed by confocal microscopy. HepG2 cells were incubated with varying concentrations of andrographolide, or with vehicle only or not treated and then infected with CHIKV E1: 226VT followed by incubation under standard conditions in the presence or absence of the drug or vehicle as appropriate. At 24 h.p.i. cells were fixed and stained to show the nucleus (red) and CHIKV E2 protein (green). Cells were examined under an Olympus FluoView 1000 confocal microscope with 60X magnification. Representative, non-contrast adjusted merged images are shown.

HepG2 cells were treated with various concentrations of andrographolide (range 1 μM to 100 μM) for 1 hr, in parallel with control treated cells (0.1% DMSO) before mock infection or infection with an MOI of 10 of CHIKV ECSA genotype E1:226VT or E1:226AS and incubated for 24 hours in the presence or absence of the compound as appropriate. Supernatants were collected for determination of titer by standard plaque assays. In addition, cells infected with E1:226VT were counted using trypan blue or analyzed by flow cytometry to determine percentage infection. A parallel experiment with E1: 226VT was undertaken with HepG2 cells grown on glass coverslips for analysis by confocal microscopy. Results showed a dose-dependent reduction of the number of infected cells, with 80% inhibition at 100 μM and no significant level of cell death was observed by trypan blue staining (Fig. 1a). A confirmatory dose-dependent reduction in CHIKV E2 protein expression was seen in cells analyzed by confocal microscopy (Fig. 2).

The results of the plaque assays showed approximately 3log_10_ reduction of CHIKV E1:22AS viral production upon treatment with 100 μM of andrographolide (EC_50_ = 77.39 μM) as shown in Fig. 1b, while CHIKV E1:226VT showed an approximate 2log_10_ reduction with 100 μM of this compound (EC_50_ = 77.44 μM) as shown in Fig. 1c, suggesting that CHIKV E1:226AS is somewhat more sensitive to the action of andrographolide than CHIKV E1:226VT.

**Figure 3:**
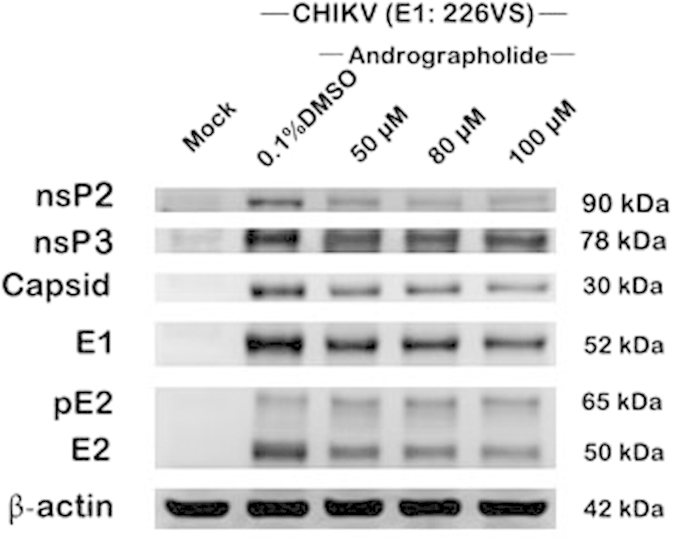
Effect of andrographolide treatment on CHIKV protein expression. HepG2 cells were incubated with varying concentrations of andrographolide or with vehicle only or not treated and then infected with CHIKV E1: 226VT or mock infected followed by incubation under standard conditions in the presence or absence of the drug or vehicle as appropriate. At 24 h.p.i. samples were examined by western blotting for the expression of CHIKV structural and non-structural proteins.

**Figure 4:**
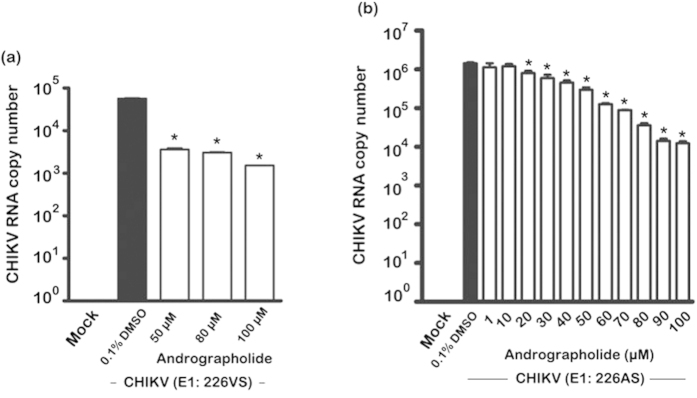
Effect of andrographolide treatment on CHIKV RNA copy number. HepG2 cells were incubated with varying concentrations of andrographolide, or with vehicle only or not treated and then mock infected or infected with (**a**) CHIKV E1: 226VT or (**b**) E1: 226AS followed by incubation under standard conditions in the presence or absence of the drug or vehicle as appropriate. At 24 h.p.i., CHIKV RNA copy number was determined by qRT-PCR. Experiments were undertaken independently in duplicate with duplicate qRT-PCR. Bars show mean +/− SD (*; *p* value < 0.05).

Using the same experimental methodology (1 hour pre-treatment and post treatment), the expression of both structural and non-structural proteins was investigated, together with the CHIKV RNA copy number. HepG2 cells treated with 50, 80 or 100 uM andrographolide and infected with E1:226VS showed a reduction in both structural (capsid, E1, pE2 and E2) and non-structural (nsP2 and nsP3) proteins (Fig. 3) as well as a reduction in CHIKV RNA copy number (Fig. 4a). A clear dose response reduction in CHIKV RNA copy number was additionally seen in HepG2 cells treated with a more comprehensive range of andrographolide and infected with CHIKV E1: 226AS (Fig. 4b).

### Time-of-addition studies

**Figure 5:**
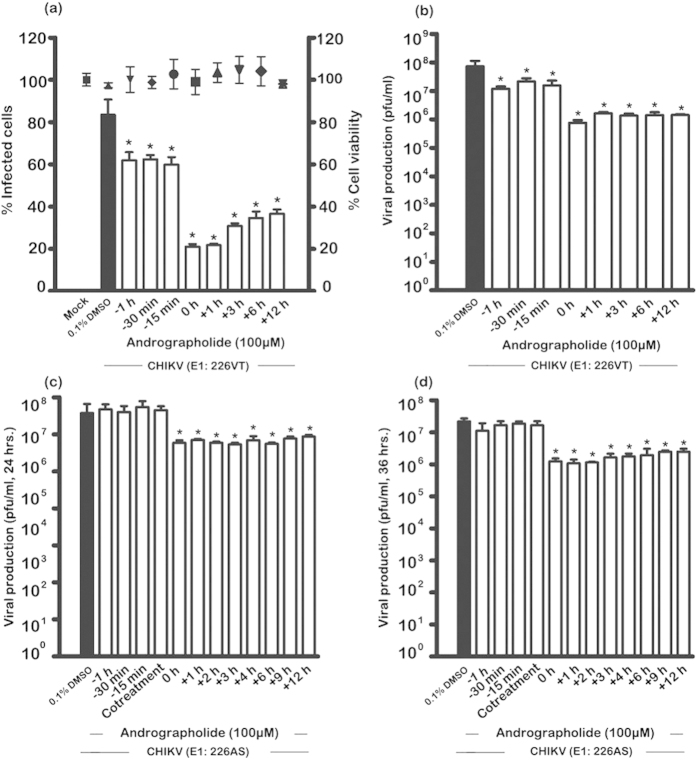
Effect of time-of-addition of andrographolide on CHIKV infection. HepG2 cells were incubated with 100 μM andrographolide or with vehicle only or not treated at the indicated time points before and after mock infection or infection with (**a**,**b**) CHIKV E1: 226VT or (**c**,**d**) CHIKV E1: 226AS. At (**a** to **c**) 24 or (**d**) 36 h.p.i. (**a**) cells were collected to determine the cell viability by trypan blue staining and infection level by flow cytometry or (**b** to **d**) supernatants were collected to determine virus titer by standard plaque assay. Experiments were undertaken independently in triplicate with duplicate plaque assay. Bars show mean +/− SD (*; *p* value < 0.05).

**Figure 6:**
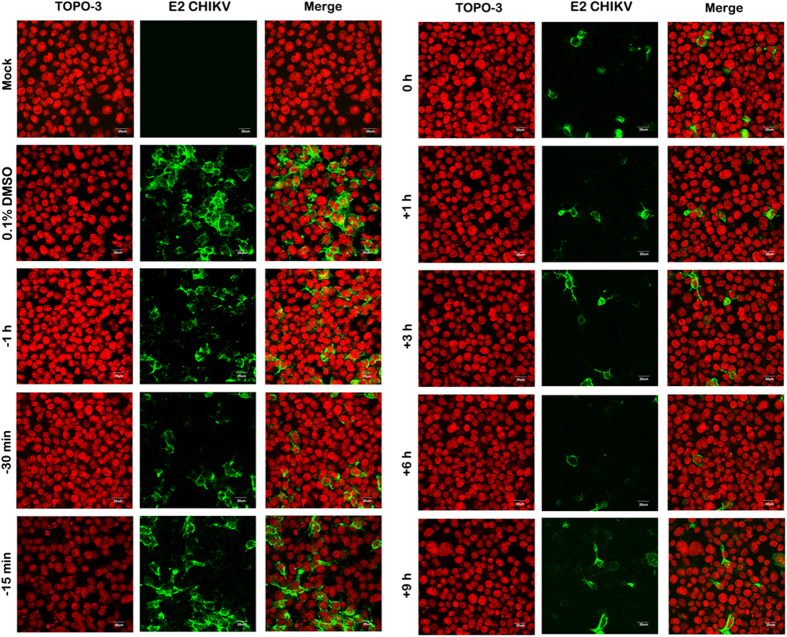
Effect of time of addition of andrographolide on CHIKV infection as assessed by confocal microscopy. HepG2 cells were incubated with 100 μM andrographolide or with vehicle only or not treated at the indicated time points before and after mock infection or infection with CHIKV E1: 226VT. At 24 h.p.i. cells were fixed and stained to show the nucleus (red) and CHIKV E2 protein (green). Cells were examined under an Olympus FluoView 1000 confocal microscope with 60X magnification. Representative, non-contrast adjusted merged images are shown.

A time-of-addition analysis was undertaken, with andrographolide used to pre-treat cells prior to infection for 1 hr, 30 min or 15 min or to post treat cells at 0, 1, 3, 6 and 12 hrs post infection. For cells infected with CHIKV E1:226VT percentage infection was determined along with cell viability and infectious virus production by standard plaque assay, while for cells infected with CHIKV E1:226AS virus production was determined at 24 and 36 hrs post infection. In a parallel experiment, cells grown on coverslips were infected with CHIKV E1:226VT and E2 expression investigated by confocal microscopy. Results (Fig. 5a) show that while some effect was seen on percentage infection for cells pretreated with andrographolide, the most significant effect was seen with the addition of andrographolide immediately after the infection period. Treatment of cells at this time point reduced levels of infection to 20% of control levels, and no significant loss of cell number was observed (Fig. 5a). This time point is similar to the previous experiment where andrographolide was added both pre- and post- infection, and the result for 100μM between the two experiments is consistent. Results of confocal microscopy (Fig. 6) showed a marked reduction in E2 expression with post-treatment, but little or no reduction in cells pre-treated with andrographolide. For viral output, again while some reduction was seen in viral titer in cells with (only) pre-treatment (Fig. 5b), the largest effect was seen for cells treated immediately after infection (0 hour time point) where again, some 2log_10_ reduction was seen in virus titer as compared to control. A further experiment to investigate the potential time that andrographolide was able to exert an effect was undertaken by increasing the pre-treatment time. An effect on infection could be seen at 12 hours pre-treatment, but not at 24 hours pre-treatment, implying that andrographolide was capable of exerting an effect for up to 12 hours (Supplemental Figure S4).

For cells infected with CHIKV E1:226AS, no significant reduction was seen in virus titer with pre-treatment of the cells (Fig. 5c,d), and significant reductions in titer were only observed with post infection treatment at both 24 (Fig. 5c) and 36 (Fig. 5d) hrs post infection. Collectively, the results suggest that andrographolide has minimal effect on the CHIKV entry process, but exerts its effect at a post entry stage.

**Figure 7:**
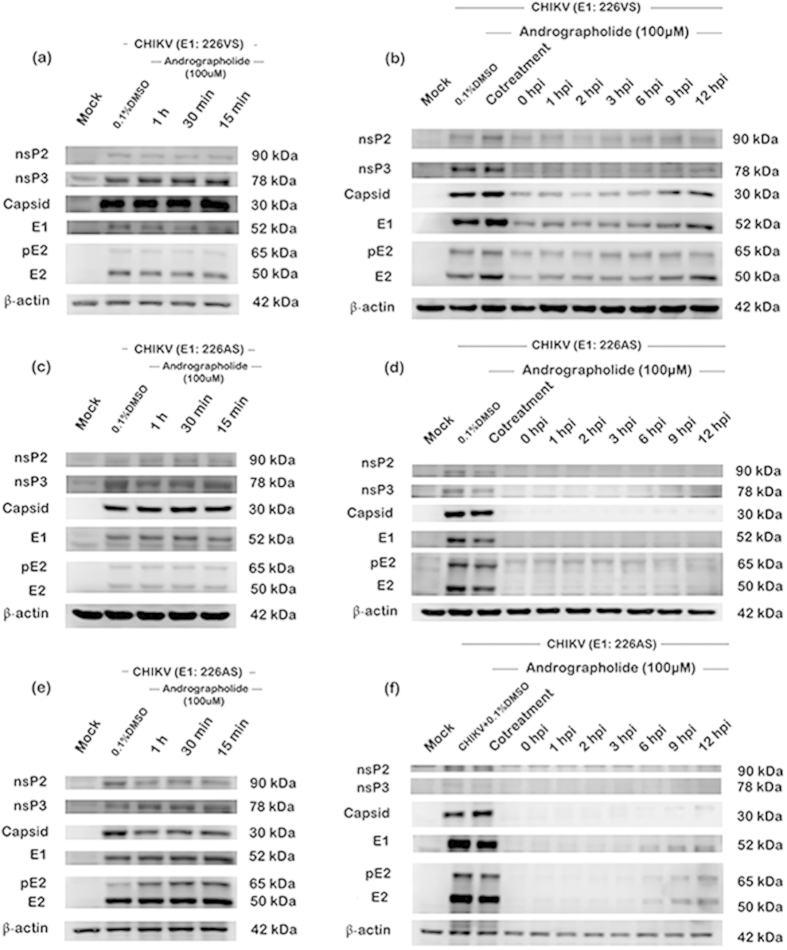
Effect of time-of-addition of andrographolide on CHIKV protein expression. HepG2 cells were incubated with 100 μM andrographolide or with vehicle only or not treated at the indicated time points before (**a**,**c**,**e**) or after (**b**,**d**,**e**) mock infection or infection with CHIKV: E1 226VS (**a**,**b**) or E1: 226AS (**c** to **f**) and at a time point of 24 (**a** to **d**) or 36 (**e**,**f**) hours after infection the expression of structural (Capsid, E1, pE2 and E2) and non-structural (nsP2, nsP3) CHIKV proteins together with actin as a control was analyzed by western blotting. Experiments were undertaken independently in duplicate.

Evaluation of structural and non-structural protein expression in the time-of-addition studies showed little or no reduction of CHIKV protein expression under conditions of andrographolide pre-treatment for both CHIKV E1:226VS and E1:226AS (Fig. 7), but reduction in both structural (capsid, E1, pE2 and E2) and non-structural (nsP2 and nsP3) protein expression was observed for both strains under conditions of post-treatment (Fig. 7). The reduction in protein expression was markedly greater for E1: 226AS, consistent with the previous observations of a greater sensitivity of this strain towards andrographolide. Surprisingly, even extending the period of culture from the standard 24 hours p.i. (Fig. 7c,d) to 36 h.p.i. (Fig. 7e,f) showed little CHIKV protein expression, although some increase in the expression of E1, pE2 and E2 was seen with the later time-of-additions when analysis was conducted at 36 h.p.i. (Fig. 7e,f). The blots for the independent duplicate analysis for E1:226AS at 24 and 36 hours are shown in Supplemental Figure 5.

**Figure 8:**
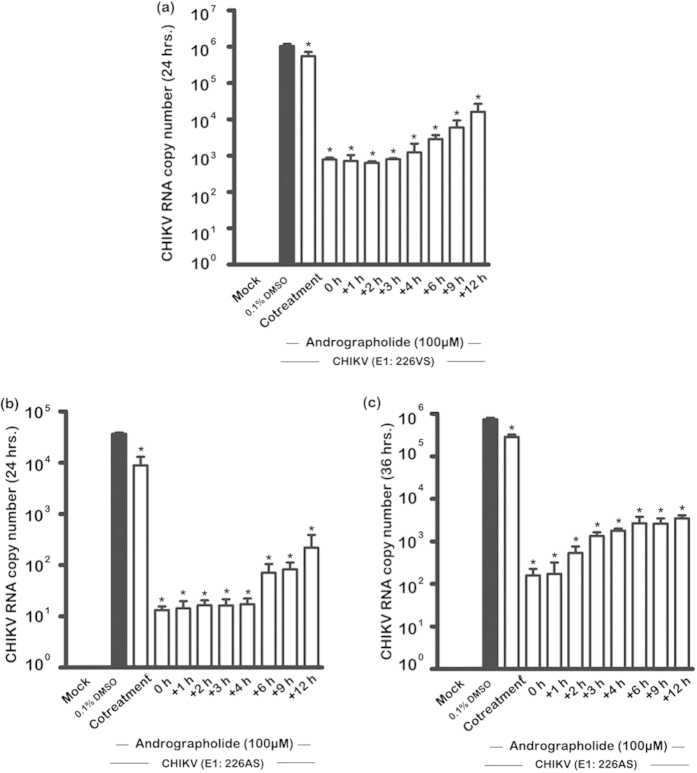
Effect of time-of-addition of andrographolide on CHIKV RNA copy number. HepG2 cells were incubated with 100 μM andrographolide or with vehicle only or not treated at the indicated time points before or after mock infection or infection with CHIKV: E1 226VS (**a**) or E1: 226AS (**b**,**c**). At 24 (**a**,**b**) or 36 (**c**) hours after infection CHIKV RNA copy number was quantitated by qRT-PCR. Experiments were undertaken in duplicate with duplicate qRT-PCR. Bars show mean +/− SD (*; *p* value < 0.05).

Investigation of CHIKV RNA copy number by RT-PCR with both E1: 226VS and E1:226AS showed very marked loss of genome copy number with post-infection addition of andrographolide (Fig. 8). Copy number at 24 hours p.i. showed a 3 log reduction in genome copy number when andrographolide was added immediately after infection (0 hour) or in the following 3 or 4 hours, after which the copy number increased with increasing time-of-addition for both E1:226VS and E1:226AS (Fig. 8a,b), and the reduction was still significant when copy number was determined at 36 h.p.i. (E1: 226AS; Fig. 8c).

For time-of-addition studies evaluating both protein expression (Fig. 7) and RNA copy number (Fig. 8), one additional control was included, namely “co-treatment”. In these experiments the andrographolide was added during the 1.5 hr virus infection step. Interestingly, both protein expression studies (Fig. 7) and RNA copy number evaluation (Fig. 8) showed a clear and extremely significant difference between “co-treatment” and the “0 hour” time point (in which the infection step is compete, and the drug added immediately after this stage, before continued incubation in complete media). This result clearly demonstrates that the activity of the drug is largely confined to post virus attachment and entry processes of CHIKV.

### Effect of andrographolide at the post virus entry stages

**Figure 9:**
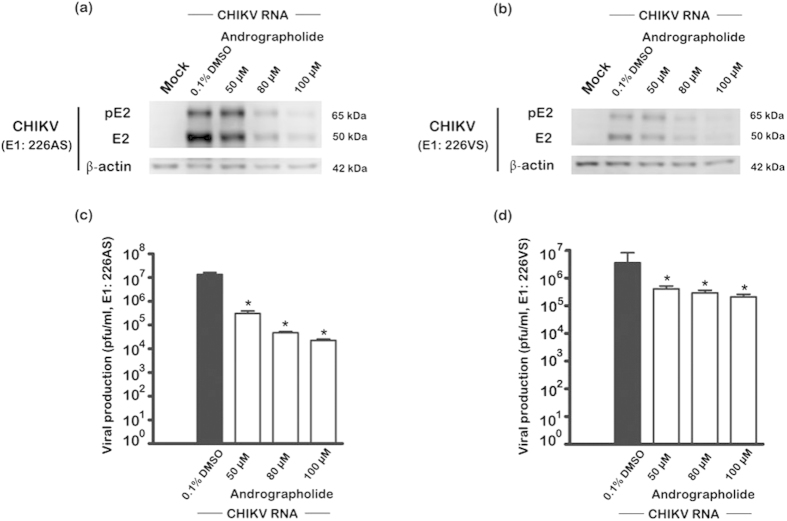
Effect of andrographolide treatment on CHIKV E1: A226 RNA transfection. HepG2 cells were directly transfected with CHIKV E1: 226AS (**a**,**c**) or E1: 226VS (**b**,**d**) RNA or mock transfected and then treated with varying concentrations of andrographolide or vehicle or not treated. At 24 h.p.i., (**a**,**b**) cellular expression of E2/pE2 and actin were determined by western blotting and (**c**,**d**) titer of CHIKV in the supernatants were determined by standard plaque assay. Experiments were undertaken independently in duplicate with duplicate of plaque assay. Bars show mean +/− SD (*; *p* value < 0.05).

To confirm that andrographolide acts on the post-entry step, cells were directly transfected with CHIKV RNA, effectively bypassing the viral entry and uncoating stages. HepG2 cells were transfected with E1:226VS and E1:226AS separately, and cultured in the presence or absence of andrographolide for 24 h.p.i. after which structural protein expression (pE2 and E2) and CHIKV titers in the supernatants were determined. Results showed reduced protein expression and significantly decreased virus titer in the presence of andrographolide (Fig. 9).

### Effect of andrographolide in BHK21 cells

**Figure 10:**
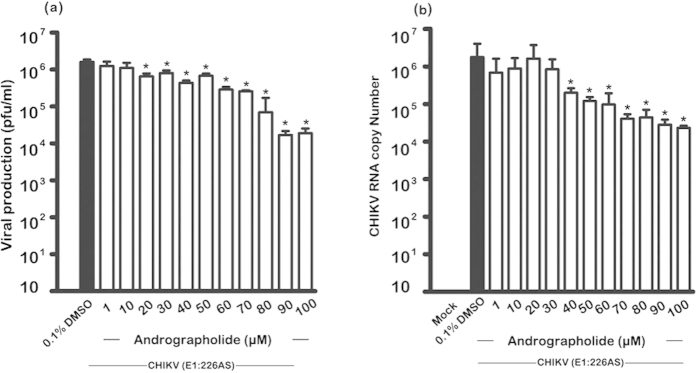
Effect of andrographolide treatment on CHIKV infection of BHK21 cells. HepG2 cells were pre-treated with varying concentrations of andrographolide or with vehicle only or not treated and then infected or mock infected with (**a**) CHIKV E1: 226VT (**b**) CHIKV E1: 226AS and (**c**) E1: 226VT followed by incubation under standard conditions in the presence or absence of the drug or vehicle as appropriate. At 24 h.p.i. (**a**) the level of viral production was determined by standard plaque assay and (**b**) the genome copy number in cells was determined by qRT-PCR. The experiments were undertaken independently in duplicate with duplicate plaque assay or qRT-PCR as appropriate. Bars show mean +/− SD (*; *p* value < 0.05).

To determine whether the effect of andrographolide was specific for liver cells, further experiments were undertaken in BHK21 cells. These cells were evaluated for cytotoxicity of andrographolide prior to use, and no cytotoxicity was observed (Supplemental Figure 6). The treatment protocol for andrographolide was consistent with the earlier studies, with 1 hour pre-treatment of the drug and the presence of the drug (range 1μM to 100 μM) media post infection. As CHIKV E1:226AS has been shown to have slightly greater sensitivity to andrographolide, this strain was selected for analysis, and both virus titer and CHIKV RNA copy number were determined after 24hrs. Results showed that both virus titer and CHIKV RNA copy number were reduced in a dose dependent manner (Fig. 10), although virus titer showed a maximal 2log_10_ reduction at 100 μM, somewhat lower than seen in HepG2 cells. The EC_50_ of andrographolide treatment in BHK21 was 88 μM, similar to the EC_50_ value of andrographolide in HepG2 cells. CHIKV RNA copy number showed a reduction consistent with the reduction seen in CHIKV titer (Fig. 10).

## Discussion

The results of this study have shown that andrographide, a bicyclic diterpenoid lactone from the traditional herb *Andrographis paniculata* (Burm. f.) Nees has potent anti-CHIKV activity. The results suggest that andrographolide exerts its activity at a post entry step, and effectively inhibits viral genome replication. Several previous studies have shown that andrographolide possesses anti-cancer^30^,^31^, anti-metastasis^32^, anti-inflammatory^33^, anti-bacterial^34^ and antiviral^35^,^36^,^37^,^38^,^39^,^40^,^41^,^42^,^43^ activities. Andrographolide has been shown to exert activity on a wide range of viruses including human papillomavirus pseudovirus^38^, Influenza A virus^37^,^43^, hepatitis B virus^36^ hepatitis C virus^39^, herpes simplex virus^41^,^42^ and human immunodeficiency virus^35^,^40^.

The mechanism by which andrographolide exerts its anti-viral activity remains unknown, although retinoic acid inducible gene-1 (RIG-1)-like receptors^43^ and the p38 MAPK/Nrf2 pathway^39^ have been proposed as possible targets for the antiviral activity against Influenza A and hepatitis C viruses respectively. However, several studies have proposed that andrographolide exerts anti-cancer activity through an effect on TLR4/NF-kB^31^,^44^,^45^,^46^, and a recent proteomic analysis identified NF-kB as a specific andrographolide binding target^47^.

TLR4 is known to activate NF-kB leading to the up-regulation of pro-IL-1β and pro-IL-18 and their subsequent processing by the Nlrp3 inflammasome and caspase 1^48^. We have previously observed the up-regulation of TLR4 and NLRP3 as well as increased processing and expression of caspase 1 in a proteomic analysis of white blood cells taken from patients suffering from different severities of chikungunya fever^49^. A recent study has also supported the activation of the inflammasome in CHIKV infection^50^. However, inflammasome activation is antiviral, and silencing of capsase 1 was shown to enhance CHIKV replication^50^. Thus andrographolide either enhances the inflammasome or is exerting its anti-CHIKV activity through an alternate pathway.

The proteomic study of targets of andrographolide also identified actin as a host cellular protein that binds andrographolide^47^. Actin has been shown to be a CHIKV binding protein^51^, and andrographolide could inhibit this interaction. While our data shows no alteration in the expression of actin, actin has been shown to be important in the transport of replication complexes from the plasma membrane to modified lysosomes in the replication of the alphavirus Semliki Forest virus^52^. However, our results suggest a very significant inhibition of protein synthesis and virus genome replication, suggesting that andrographolide may work at an earlier stage of replication.

Perhaps most interestingly, our results show that, with some strains of CHIKV, even a delayed post infection treatment with andrographolide can significantly inhibit protein expression. In particular, the western blot analysis (Fig. 7) showed that 9 or 12 hours post infection treatment almost completely abolished detectable protein expression of CHIKV E1: 226AS, and greatly inhibited expression of CHIKV E1:226VS. This result was partially confirmed by the confocal microscopy analysis which showed greatly reduced levels of CHIKV E1:226VT E2 protein at 24 hours post infection when andrographolide was added 9 hours post infection. These experiments were conducted with different virus isolates, and indeed the analyses were conducted in different countries, however, the result is remarkably consistent, and suggests that andrographolide may in some way promote the clearance of viral proteins in addition to inhibiting their production.

Several studies have shown that andrographolide is capable of inducing autophagy^53^,^54^,^55^, and autophagy has been shown to be activated in CHIKV infection^56^,^57^,^58^ where it promotes viral replication^58^. However the subversion of autophagy requires the interaction of CHIKV proteins with host cell proteins^57^, and it is possible that andrographolide interferes with the subversion process, allowing the increased activation of autophagy to promote the clearance of viral proteins rather than promoting replication. However, it has also been suggested that andrographolide also inhibits terminal autophagosome-lysosome fusion^55^ and as such disruption of autophagic flux may significantly affect CHIKV replication, with clearance of proteins occurring through an alternate pathway. In this respect, studies have shown that CHIKV infection activates the unfolded protein response^59^,^60^, a process that is known to results in increased ER-associated degradation (ERAD) activity^61^, again possibly facilitating removal of viral proteins.

While andrographolide exerted a potent anti-CHIKV effect, reducing protein synthesis and RNA genome levels, the greatest effect was seen when andrographolide was applied post entry. Both pre-treatment and co-treatment showed significantly lower levels of inhibition than post treatment. Moreover some differences in the level of inhibition were seen between different virus strains. These results would suggest that andrographolide is either targeting the CHIKV genome or proteins directly, or a protein or proteins induced by the infection. Alternatively it is possible that andrographolide is acting on multiple pathways synergistically which would be consistent with the multiple identified targets of andrographolide. Thus, andrographolide could promote an effective antiviral environment through the stimulation or inhibition of numerous targets.

Further studies are required to understand the different strain sensitivity to the action of andrographolide, but despite the variations in the strain dependent inhibition of CHIKV infection by andrographolide, a 2log_10_ inhibition of viral replication was observed with the least affected CHIKV strain, suggesting that andrographolide warrants further investigation, including studies in animal model systems, both to understand the mechanism of its inhibition of CHIKV infection, as well as to determine its suitability for clinical application. One study undertaken in mice has shown significant anti-influenza activity with therapeutic efficacy achieved at a dose of 150 mg/kg/d while LD_50_ values were > 4000 mg/kg/d suggesting promising application in anti-viral therapeutics^37^. Traditional doses of powdered *Andrographis paniculata* are believed to deliver 90–150 mg/day although higher doses have been administered^62^. However, a phase 1 dose-escalating clinical trial in HIV positive patients and volunteers was terminated at a level of 10 mg/kg bodyweight due to the occurrence of adverse effects, including an anaphylactic reaction in one patient^35^. Thus while andrographolide offers significant potential, caution must be applied in its development, and derivatives of andrographolide may offer a suitable route to safe and efficacious anti-viral therapy for CHIKV.

## Methods

### Cell lines and viruses

The human liver cell line HepG2 (ATCC Cat No. HB-8065) was cultured in Dulbecco’s modified Eagle’s medium (DMEM) supplemented with 10% heat inactivated fetal calf serum (FCS) at 37 °C with 5% CO_2._ The baby hamster kidney cell line BHK-21 (ATCC Cat No. CCL-10) was cultured in RPMI-1640 medium supplemented with 10% FCS at 37 °C with 5% CO_2_. The CHIKV strains used in this study have been previously described^29^,^63^ and consist of a CHIKV ECSA genotype: E1:226A (CHIKV-0708^29^) herein designated E1:226AS, a CHIKV ECSA genotype E1:226V (CHIKV-122508^29^) herein designated E1:226VS and a CHIKV ECSA genotype E1:226V (KP-164869^63^) herein designated E1:226VT. Virus stocks were propagated in BHK21 or C6/36 cells as previously described, and all virus titers were determined by standard plaque assay as described previously^29^,^63^.

### Andrographolide

Andrographolide (365645, Sigma) was dissolved in 100% DMSO to a final stock concentration of 100 mM and stored at −80 °C. Compound was serially diluted to various concentrations using complete DMEM. The final concentration of DMSO in media was less than 0.1%.

### Virucidal assay

Stock CHIKV ECSA genotype E1:226VT was incubated directly with vehicle only or with medium only or with different concentrations of andrographolide individually at 37 °C for 1 hr after which infectious virus titer was determined by standard plaque assay. Experiment was undertaken independently in triplicate with duplicate plaque assay.

### Cell viability and number assays

HepG2 cells were cultured in ninety-six well-tissue culture plates under standard conditions until the cells reached 90% confluence. The cell culture medium was removed and then incubated with various concentration of andrographolide (1–100 μM) diluted in complete medium with FBS prior to being cultured for 24 hrs under standard conditions before analysis using either the Vybrant® MTT Cell proliferation assay kit (V13154, Invitrogen) or the alamarBlue (Thermo Fischer Scientific, Waltham, MA) assay kit according to the manufacturers recommendations. Values were determined from 8 (MTT assay) or 3 (alamarBlue) independent replicates. Negative controls (only media and 0.1% DMSO) and positive controls (5% DMSO) were included. To determine cell number, cell cultures were trypsinized by the addition of 0.25%Trypsin/0.1%EDTA for 3 min and then cell suspensions were diluted with 0.4% trypan blue solution. Cells were counted using a hemocytometer.

### Andrographolide treatment of cells

HepG2 or BHK 21 cells as appropriate were cultured in six well culture plates and then grown until the cells reached approximately 90% confluency. The cells were washed with PBS and then incubated with 0.1% DMSO (no treatment control) or with andrographolide at various concentrations (range 1 μM to 100 μM) for 1 hr followed by washing twice with PBS before infection with CHIKV at 10pfu/cell at 37 °C for 1.5 hrs in the absence of the compound. Cells were then washed three times with PBS and media supplemented with 2% FCS and containing appropriate concentration of each drug was added before being incubated under standard conditions for 24 or 36 hrs. For pre-treatment studies, cells at approximately 90% confluency were incubated with 100 μM andrographolide for 1 h, 30 min or 15 min prior to infection. Cells were washed twice with PBS and infected with CHIKV at 10 pfu/cell under standard conditions. After infection cells were washed three times with PBS before the addition of complete media (without andrographolide) and incubated under standard conditions. For post-infection treatment studies, cells were infected or mock infected as appropriate and 100 μM andrographolide was added in complete media at 0, 1, 2, 3, 4, 6, and 12 hrs after infection. Cells were incubated under standard conditions until analyzed. In co-treatment studies, cells were infected with virus in the presence of andrographolide. For all experiments, supernatant and cells were harvested at appropriate time points, and all experiments were undertaken independently in triplicate with duplicate plaque assay where appropriate. Control experiments were undertaken using 0.1% DMSO as vehicle.

### Flow cytometry

Mock- or CHIKV-infected cells (with or without compound treatment as appropriate) were harvested at appropriate time points and then incubated with 10% normal goat serum (NGS; Gibco^TM^ Invitrogen) in PBS on ice for 30 min. The cells were washed twice with 1 ml of PBS followed by fixing with 200 μl of 4% paraformaldehyde in PBS at room temperature in the dark for 20 min. After washing twice with 1 ml of 1% BSA in PBS, the cells were permeabilized with 200 μl of 0.2% saponin in PBS for 10 min. Subsequently, the cells were washed twice with 1 ml of 1% BSA in PBS followed by overnight incubation with 50 μl of a mouse monoclonal anti-alphavirus (3581) antibody (STSC-58088, Santa Cruz Biotechnology, Santa Cruz, CA) diluted 1:200 in 1% BSA in PBS with constant agitation at 4 °C. After washing 5 times with 1 ml of 1% BSA in PBS, the cells were incubated with 25 μl of a goat anti-mouse IgG conjugated with fluorescein (02-18-06; KPL, Guilford, UK) diluted 1:20 in 1% BSA in PBS for 1 hr with constant agitation at room temperature in the dark. The cells were then washed 5 times with 1 ml of 1% BSA in PBS and resuspended in 200 μl of PBS. The fluorescence signal was analyzed by flow cytometry on a BD FACalibur cytometer (Becton Dickinson, CA, USA) using CELLQuest™ software. All experiments were undertaken independently in triplicate.

### Indirect immunofluorescence

Indirect immunofluorescence was undertaken as described previously^63^. Briefly mock- or CHIKV-infected HepG2 cells grown on glass coverslips were washed three times with PBS and fixed with 100% ice-cold methanol at room temperature for 15 min. The coverslips were kept at −20 °C until use. After washing twice with PBS, the cells were blocked with 10% normal goat serum (NGS; Gibco Invitrogen) for 1 hr at 4 °C and then washed twice with PBS. Subsequently, cells were permeabilized with 0.3% Triton X-100/PBS for 10 min and washed twice with 0.03% Triton X-100/PBS at room temperature with gentle agitation for 5 min. The cells were incubated with a mouse monoclonal anti-alphavirus (3581) antibody (STSC-58088, Santa Cruz Biotechnology) diluted 1:100 in PBS at 4 °C overnight followed by washing four times with 0.03% Triton X-100/PBS. The cells were incubated with a Fluorescein isothiocyanate (FITC) conjugated goat anti-mouse IgG antibody (02-18-06; KPL, Guilford) and To-Pro3 (Molecular Probes, Life Technologies, Grand Island, NY) diluted 1:200 in PBS at room temperature in the dark for 1 hr follow by washing six times with 0.03% Triton X-100/PBS. The cover slips were mounted onto glass slides using Prolong® Gold antifade reagent (Invitrogen) before visualization under an Olympus FluoView 1000 (Olympus Corporation, Shinjuku-ku, Tokyo) with a 60x objective lens and equipped with Olympus FluoView Sofeware v. 1.6.

### Western blotting

Cells (HepG2 or BHK21) were lysed with the addition of 200 μl M-PER Mammalian Protein extraction reagent (Thermo Scientific, Lafayette, CO) containing protease inhibitor cocktail and EDTA at 4 °C for 10 min. Cells were then scraped and collected in microcentrifuge tube before being centrifuged at 10,000 x g for 10 min at 4 °C. The supernatant was transferred to a new tube and the protein concentration determined using the Bradford assay (Bio-Rad, San Francisco, CA). Protein solutions were kept at −80 °C until use. The proteins were separated by electrophoresis through 10% polyacrylamide gels run at 100 V for 3 hrs. The PageRuler prestained protein ladder (Fermentas) was used as a molecular weight standard. Proteins were transferred to nitrocellulose membranes using the Bio-Rad semidry transfer system (Bio-Rad).

Membranes were blocked overnight at 4 °C using either 5% skim milk dissolved in TBS-T (CHIKV E2 western analysis) or 5% BSA in TBS-T (all other antibodies). Blots were washed three times with PBS prior to incubation with a 1:50 dilution of an in house anti-nsP2 rabbit polyclonal antibody, a 1:50 dilution of an anti-nsP3 rabbit polyclonal antibody^29^, a 1:300 dilution of an in house anti-capsid rabbit polyclonal antibody^29^, a 1:200 dilution of an in house anti-CHIKV E1 rabbit polyclonal antibody, 1:100 dilution of an anti-CHIKV E2 rabbit polyclonal antibody^29^ or a 1: 10,000 dilution of an anti-actin mouse monoclonal antibody (MAB 1501; Millipore, Temecula, CA). Blots were subsequently incubated with a 1:10,000 dilution of a horseradish peroxidase conjugated polyclonal goat anti-rabbit IgG (31460; Thermo Scientific, Rockford, IL) or a 1:10,000 dilution of a horseradish peroxidase conjugated polyclonal goat anti-mouse IgG (31430; Thermo Scientific, Rockford, IL). The signals were detected using SuperSignal West Dura Chemiluminescent Substrate (Thermo Scientific, Hudson, NH). Western blots were undertaken independently in duplicate.

### Viral RNA transfection

CHIKV RNA was extracted from virus stock using the QIAamp viral RNA minikit (Qiagen GmbH) according to the manufacturers’ protocol and the concentrations of RNAs were measured by spectrophotometry. A total of 200 ng CHIKV E1:226AS or E1:A226VS RNA diluted in 25 μl DharmaFECT cell culture reagent (B004500, Thermo Scientific) was mixed with 0.4 μl of DharmaFECT transfection reagent (T2001, Thermo Scientific) and 24.6 μl DharmaFECT cell culture reagent and incubated at room temperature for 30 min to allow complexes to form. HepG2 cells were seeded on 24 well plates and then grown until the cells reached approximately 90% confluency. HepG2 monolayers were washed once with PBS and incubated with the complex mixture for 1.5 hrs at 37 °C with gentle rocking every 10 min. Andrographolide (final concentrations 50, 80, and 100 μM) in parallel with vehicle diluted in DMEM with 10% FCS was added into the wells. The cells were incubated for 24 hrs at 37 °C with 5% CO_2_ and the cells and supernatants were harvested for further analysis. The experiments were undertaken independently in duplicate with duplicate plaque assay.

### Quantitative reverse transcription-PCR (qRT-PCR)

Total RNA was extracted using the RNeasy minikit (Qiagen) according to the manufacturer’s protocol. A standard curve was generated using serial dilutions of viral RNAs. First strand of cDNA was synthesized using M-MLV Reverse Transcriptase (M170B, Promega) and gene specific primer CHIKV_CAP R. Following first-strand synthesis, samples were subjected to amplification in a 25 μl reaction mixture containing 12.5 μl of Maxima SYBR Green/ROX qPCR Master mix (K0221, Thermo Scientific), 0.5 μl of forward and reverse primers (capsid or actin), 2 μl of cDNA and 9.5 μl of nuclease-free water. Reactions were carried out in the Applied Biosystems StepOnePlus real-time PCR system (Applied Biosystems, Carlsbad, CA), beginning with 40 amplification cycles at 95 °C for 3 s each and 60 °C for 1 s for fluorescence measurement during amplification. Following amplification, a melting curve analysis was performed at 95 °C for 15 s and 60 °C for 1 min to verify the melting temperatures of PCR products amplified by the primer pairs.

The sequences of primers were as follows:

CHIKV_CAP F: 5′ GCGGTACCCCAACAGAAG 3′

CHIKV_CAP R: 5′ GGTTTCTTTTTAGGGTGGCTG 3′

ACTIN F: 5′ AGCGCGGCTACAGCTTCA 3′

ACTIN R: 5′ GGCGACGTAGCACAGCTTCT 3′

The copy numbers of CHIKV RNA were calculated from the cycle threshold value of the amplification plot using the standard curve. The experiment was undertaken independently in duplicate with duplicate qRT-PCR.

### Statistical analyses

All data were analyzed using the GraphPad Prism program (GrapPad Software). Statistical analysis of significance was undertaken by ANOVA with Tukey’ post hoc comparisons on raw data reads using SPSS, p < 0.05 for significance (SPSS Inc., Chicago, IL, USA). CC_50_ and EC_50_ values were calculated using the freeware ED50plus (v1.0) software (http://sciencegateway.org/protocols/cellbio/drug/data/ed50v10.xls).

## Additional Information

**How to cite this article**: Wintachai, P. *et al*. Activity of andrographolide against chikungunya virus infection. *Sci. Rep*. **5**, 14179; doi: 10.1038/srep14179 (2015).

## Supplementary Material

Supplementary Information
