# Supplementary material for: Activity of andrographolide against chikungunya virus infection
Source: Sci Rep. 2015 Sep 18;5:14179. doi: 10.1038/srep14179 (PMC4585663; doi:10.1038/srep14179)
Supplement: Supplementary Information [file srep14179-s1.pdf]

Activity of andrographolide against chikungunya virus infection

Phitchayapak Wintachai, Parveen Kaur, Regina Ching Hua Lee, Suwipa Ramphan, Atichat Kuadkitkan, Nitwara Wikan, Sukathida Ubol, Sittiruk Roytrakul, Justin Jang Hann Chu and Duncan R. Smith

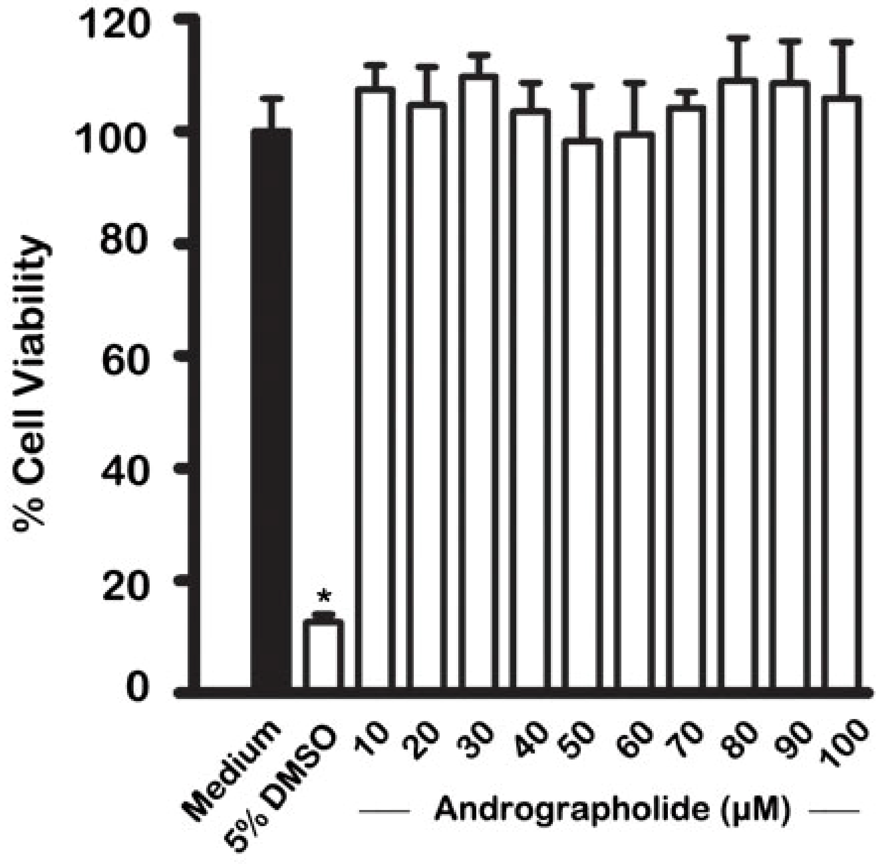

Figure S1 Determination of andrographolide cytotoxicity to HepG2 cells: MTT assay

HepG2 cells were incubated with different concentrations of andrographolide or not treated for 24 hours followed by MTT cell viability assays. Data is derived from 8 replicates. Treatment with 5% DMSO was used as a positive control. Bars show mean +/- SD (*; *p* value <0.05).

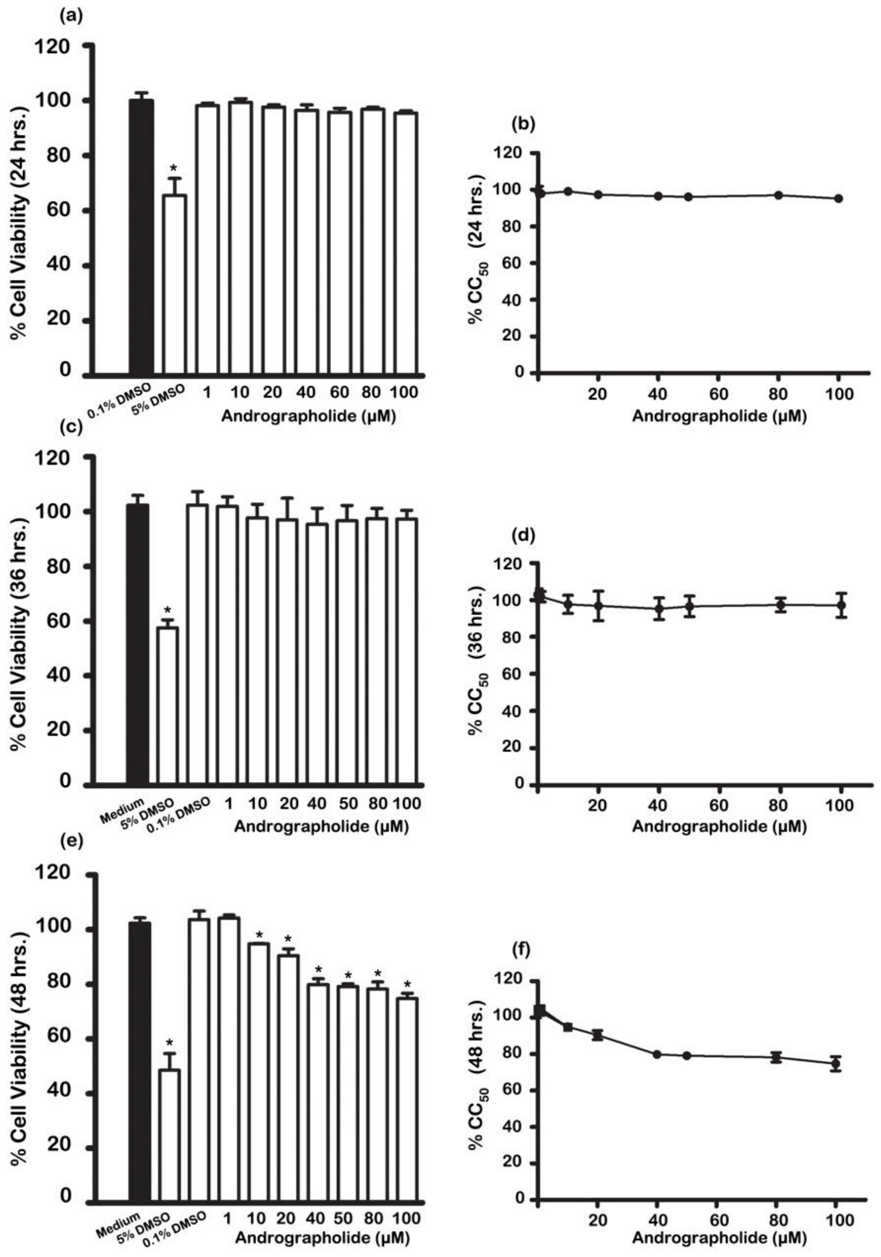

Figure S2 Determination of andrographolide cytotoxicity to HepG2 cells: alarmar Blue

HepG2 cells were incubated with different concentrations of andrographolide or vehicle or not treated for (a) 24, (c) 36 or (e) 48 hours followed by alamar Blue assay. Subsequently, the data was used to calculate 50% cell cytotoxicity values at (b) 24 (d) 36 and (e) 48 hours Experiments were undertaken in triplicate. Treatment with 5% DMSO was used as a positive control. Bars show mean +/- SD (*; *p* value <0.05).

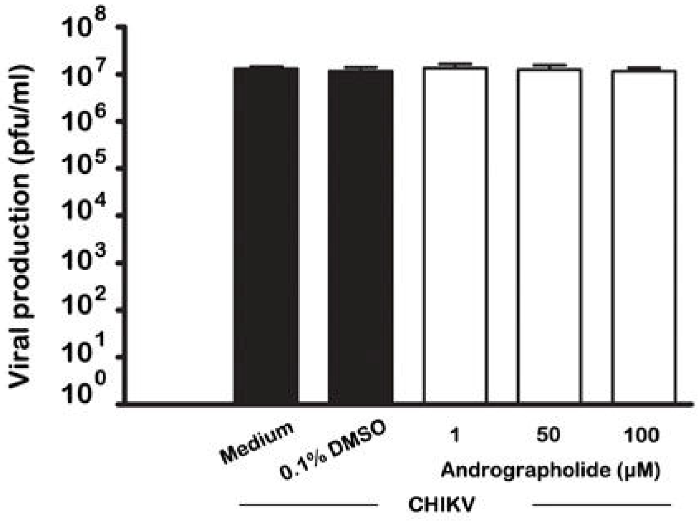

Figure S3 Evaluation of virucidal activity of andrographolide

Stock CHIKV (ECSA E1:226VT) was incubated at 37°C for 1 hr with medium only, vehicle only or different concentrations of andrographolide before determination of viral titer by standard plaque assay. Experiment was undertaken independently in triplicate, with duplicate plaque assay. Bars show mean +/- SD.

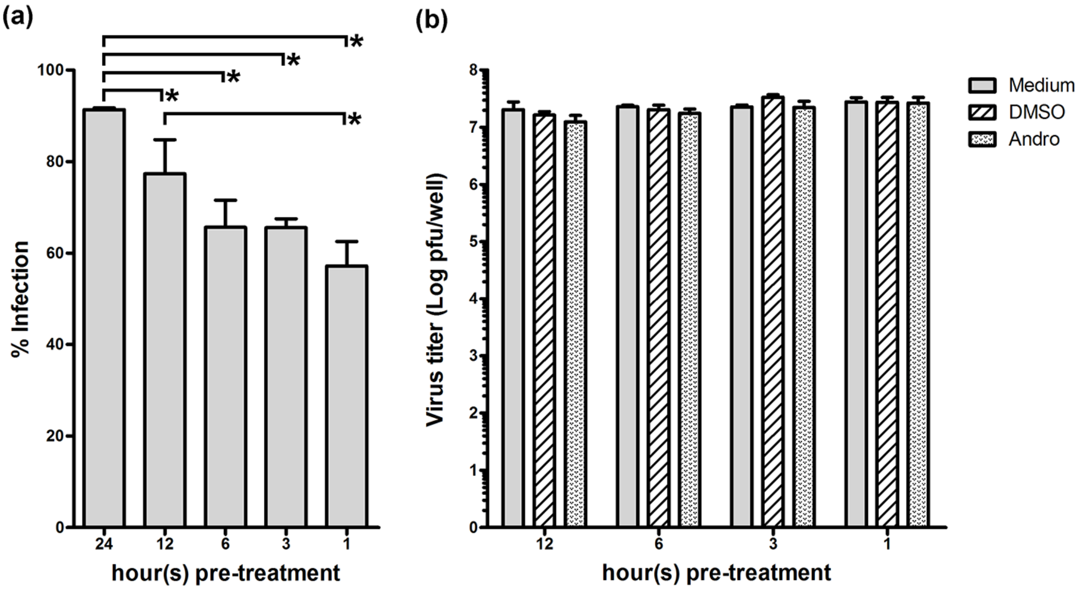

Figure S4 Effects of extended incubation with andrographolide

(a) HepG2 cells were pre-incubated with 100 μM andrographolide for the indicated times before infection with CHIKV E1: 226VT and at 24 h.p.i cells were collected to determine the infection level by flow cytometry. Experiment was undertaken independently in triplicate with duplicate plaque assay. Bars show mean +/- SD (*; *p* value <0.05).

(b) Stock CHIKV (ECSA E1:226VT) was incubated at 37°C for differing times with medium only, vehicle only or with 100 μM andrographolide before determination of viral titer by standard plaque assay. Experiment was undertaken independently in triplicate, with duplicate plaque assay. Bars show mean +/- SD.

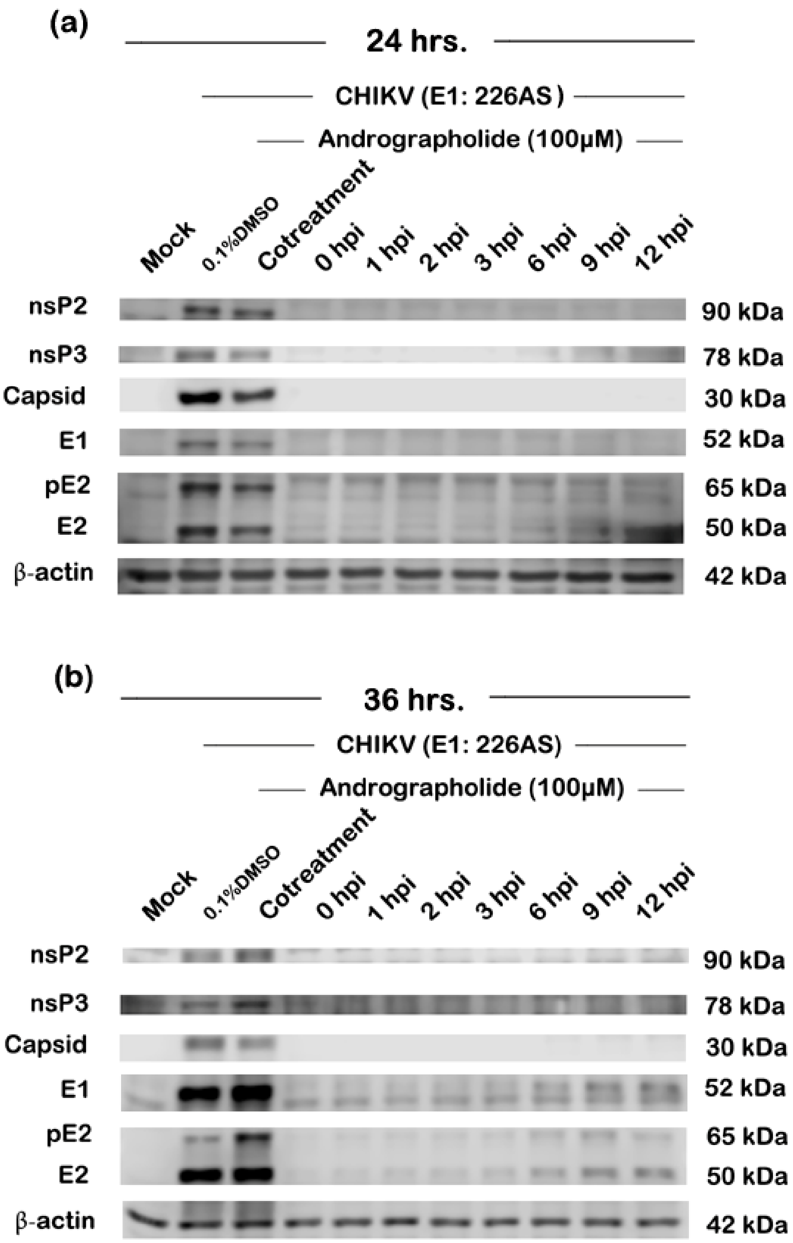

Figure S5. Duplicate western blot results of Figure 7 Panels (d) and (e).

Refer to Figure 7 for experimental details.

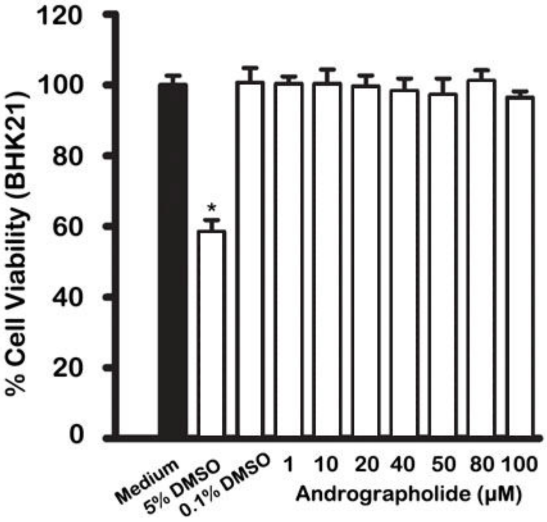

Figure S6 Determination of andrographolide cytotoxicity to BHK21 cells: alamar Blue

BHK21 cells were incubated with different concentrations of andrographolide or vehicle or not treated for 24 hours followed by alamar Blue assay. Experiments were undertaken in triplicate. Treatment with 5% DMSO was used as a positive control. Bars show mean +/- SD (*; *p* value <0.05).
